# Five Steps for the Maintenance and Interception of Complications in Zygomatic Implants

**DOI:** 10.3390/dj11100226

**Published:** 2023-09-22

**Authors:** Consuela Sanavia, Edoardo Vallerga, Fanny Alessi, Tiziano Tealdo, Marco Bevilacqua, Christian Alberti, Maria Menini, Paolo Pesce

**Affiliations:** 1 Department of Surgical Sciences, University of Genoa, 16126 Genoa, Italy; consuela1@icloud.com (C.S.); tiziano@tealdocentriodontoiatrici.com (T.T.); m.bevilh2o@icloud.com (M.B.); 2 Independent Researcher, 12058 Santo Stefano Belbo, Italy; edoardo@tealdocentriodontoiatrici.com; 3 Independent Researcher, 36027 Rosà, Italy; fanny.alessi@gmail.com (F.A.); studiodentisticoalberti@gmail.com (C.A.); 4 Division of Prosthodontics and Implant Prosthodontics, Department of Surgical Sciences, University of Genoa, 16126 Genoa, Italy; maria.menini@unige.it

**Keywords:** dental implants, zygomatic implants, maintenance, dental hygienist, guidelines

## Abstract

Zygomatic implants are used for the rehabilitation of the upper jaw of patients with severe and moderate bone atrophy. Possible post-surgical complications include soft tissue dehiscence, sinusitis, and prosthodontic fractures, and maintaining an accurate control is crucial. Additionally, zygomatic implants have a unique peri-implant anatomy, making traditional periodontal parameters unsuitable. The present paper aims to provide guidelines for the maintenance and interception of complications in patients rehabilitated with these kinds of implants. The proposed protocol includes: 1. intra/extraoral and temporo-mandibular joint examination; 2. soft tissue and transmucosal path observation with magnifiers; 3. peri-implant health indices and digital stimulation of tissues; 4. examination of prosthodontic devices; and 5. photographic recording. These steps facilitate the comprehensive evaluation and monitoring of clinical conditions of zygomatic-supported rehabilitations, including dehiscence and occlusal wear during follow-up appointments.

## 1. Introduction

Zygomatic implants are increasingly being used to treat patients with severe bone atrophies of the upper jaw. While this technique has a success rate comparable to conventional techniques, it is not without complications and requires a certain level of surgical experience [1,2].

Zygomatic implants have demonstrated their effectiveness as a viable solution in addressing the challenges of managing atrophic edentulous maxilla [3,4,5,6] and maxillectomy defects [7]. Brånemark introduced these implants to cater to patients requiring prosthetic rehabilitation due to extensive maxillary defects resulting from tumor resections, trauma, and congenital issues [8]. The zygomatic arch’s bone was utilized as a secure foundation for a lengthy implant, which, when combined with traditional implants, could serve as an anchor for epistheses, prostheses, and/or obturators. This innovative approach has significantly enhanced the rehabilitation prospects for these patients, restoring both function and aesthetic appeal. Moreover, it has granted numerous individuals the opportunity to reclaim a sense of normalcy in their social lives.

Appropriate maintenance is fundamental for long-term success and the prevention of complications of dental implants. Renvert et al., during the 2017 world workshop, reported on how to define a dental implant as healthy [9]. However, zygomatic implants present some unique characteristics that differentiate them from classical implants (length; inclination; position; relationship with other body structures such as the sinus and eye; absence or reduced amount of bone in the crestal area, prosthesis design) and that make the classical examination approach impossible.

A recent systematic review highlighted soft tissue dehiscence, sinusitis, and prosthodontic fractures as the most common complications affecting rehabilitations supported by zygomatic implants [4]. Additionally, during maintenance, severe gingival hyperplasia/hypertrophy, pressure ulcers, fistulas, and horizontal gingival fissures may be observed. Aparicio et al., in 2006, also suggested that sinus health control should be performed as part of the maintenance program [10].

Soft tissue alterations may increase the risk of bacterial mucositis, and preventing and controlling this is crucial to prevent peri-implantitis [11]. Indeed, although a direct correlation between plaque accumulation and peri-implantitis has not been demonstrated for full-arch implant-supported rehabilitations [12,13], microbiological studies on zygomatic implants show a correlation between bleeding on probing and periodontal pathogenic bacteria [14].

Zygomatic implants have a different peri-implant anatomy compared to standard intraoral implants, since the vestibular portion of the implant is surrounded by soft tissues only most of the time, and sometimes the palatal bone is also missing, as implant stability is mainly given by the implant apex inserted into the zygoma. This prevents the use of standard periodontal parameters usually applied for dental implants and requires the use of a delicate probing technique to avoid altering desmosomal adhesion. Probing will only serve to verify the good soft tissue attachment and prevent complications, as suggested in the prospective study by Agliardi et al. in 2017 [15]. Additionally, the emergence of the implant often diverges from the bone crest, either buccally or palatally, and the formation of mucosal folds, the lack of adherent gingiva, and the frequent palatal placement make the examination, probing and peri-implant hygienic maneuvers difficult when the prosthesis is in situ. Although probing around zygomatic implants is challenging, it remains one of the most useful clinical acts to intercept peri-implant tissue inflammation.

According to Aparicio et al., the reporting of results and complications pertaining to zygomatic implants in the existing literature exhibits inconsistency and lacks a standardized systematic review. Notably, there exists a dearth of clear criteria tailored to precisely delineate outcomes in zygomatic implant rehabilitation and to gauge the degree of success or potential treatment risks associated with them. Furthermore, a tendency persists to appraise and analyze zygomatic implants on the same grounds as conventional implants placed in pristine alveolar bone. Nonetheless, zygomatic implants deviate from traditional implants in biomechanics, clinical protocols, outcomes, and eventual complications. These implants are linked to resorptive changes in both the alveolar and the basal bone, rendering the application of conventional assessment criteria inadequate for describing implant outcomes [16].

Considering implant–prosthodontic rehabilitation as a whole, a careful observation of prosthodontic structures is also necessary to detect fracture lines or abnormal wear to report to the prosthodontist. An examination of the temporomandibular joint (TMJ) should also be routinely performed to intercept any occlusal changes or patient-reported pain. In 2020, Aparicio revised the zygoma success code, updating the ORIS criteria of success [16]. Based on Aparicio’s criteria, the evaluation of zygomatic implants can be categorized into five possible conditions:

- Success Condition 1: This represents the optimal stage, where the zygomatic implant shows excellent performance and meets all the proposed criteria for success.
- Success Condition 2: This condition indicates a minor alteration from the routine, without any significant clinical impact on the implant’s functionality or patient’s well-being.
- Success Condition 3: In this situation, the zygomatic implant shows borderline characteristics with clinically manifested alterations. However, these alterations can still be successfully treated to ensure the implant’s long-term viability.
- Condition 4: This condition refers to a surviving implant that supports the prosthesis, but it has not been measured using the proposed evaluation criteria. Further assessment may be necessary to determine its overall success.
- Condition 5: This reflects implant failure, where the zygomatic implant has not met the expected criteria for success and requires appropriate intervention or revision.

It is important to assess zygomatic implants thoroughly, using these criteria to ensure the best outcomes for patients and to identify any potential issues that may arise during treatment. No specific guidelines for the examination of zygomatic implants preventing possible complications are present in the literature. This is the first paper aiming to provide guidelines for maintenance and the interception of possible complications of zygomatic implants, allowing the early identification of patients in stage 2 and 3 of the ORIS scale. The clinical procedures and devices useful for a comprehensive extra- and intra-oral examination will be described for the prevention or early interception of biological and technical complications in patients with severe bone atrophy who have undergone advanced implant–prosthodontic rehabilitations with zygomatic implants.

## 2. Materials and Equipment

The proposed protocol is intended for fixed rehabilitations supported by zygomatic implants and has been developed on the basis of the clinical experience of the authors in the rehabilitation and maintenance of more than 200 patients rehabilitated with zygomatic implants.

At each follow-up appointment, the clinician must first of all carefully check the clinical record and the radiographic exams realized following the placement of the prosthesis. This will enable them to ascertain the number of connected implants, their positions, connections, and transmucosal paths, and prosthesis fitting and design.

In this type of rehabilitation, it would also be advisable to have a comprehensive set of photographs of the mouth taken after implant insertion and without the prosthesis, as a baseline reference for the comparison of future observations.

At each follow-up appointment, before intra- and extra-oral examination, the clinician should interview the patient to obtain information about his/her satisfaction with the oral rehabilitation and to identify possible symptoms and to check adherence to oral hygiene instructions.

## 3. Detailed Procedure

After implant insertion, a provisional full-arch screwed prosthesis is delivered in the following 24 h. The authors believe that a provisional prosthesis is needed for the first 4 months to achieve the stabilization of bone and soft tissues, especially considering the significant tissue detachment often required during the intervention.

Surgical sutures are removed two weeks after surgery, and during the healing phase the patient should be advised to use an extra-soft toothbrush with a gel-based toothpaste containing 0.12–1% chlorhexidine digluconate [17] and a gel or mouthwash with hyaluronic acid to improve the healing of mucosal tissues.

During the provisional phase it is suggested to check on the patient at least once a month.

Four months after surgery, a radiographic control is performed, a final impression is obtained, and a definitive prosthesis is delivered. The final implant-supported prosthesis should be tailored to prevent plaque accumulation, provide access for the patient’s home hygiene, and allow for clinical evaluation.

It is recommended to schedule four appointments with the dental hygienist during the first year. In the first appointment with the dental hygienist, one month after receiving the definitive prosthesis, the dental hygienist educates the patient on proper hygiene and maintenance and starts the examination using the proposed five-step protocol. This step is essential to prevent complications related to poor patient hygiene and to detect any issue that may require specialist attention, which will be described in the following five steps.

This aspect becomes particularly important for patients with complete rehabilitations and severe atrophy. Often, patients in need of such rehabilitations have lost a considerable number of teeth due to poor oral care and inadequate oral hygiene, and may also be smokers or have limited manual dexterity. Additionally, full-arch prosthodontic structures do not resemble the natural tooth anatomy and can be bulky and challenging to clean, especially in lingual and palatal regions. Therefore, an intensive re-education program is necessary.

For these full-arch prosthodontic reconstructions, the authors recommend avoiding prosthesis removal for professional oral hygiene, except in cases of severe mucositis, severe inflammation, peri-implantitis, suppuration, swelling, or technical complications. Therefore, before delivering the definitive prosthesis, it is advisable to photograph it, also including a picture of the intaglio surface. This will help the dental hygienist to properly instruct the patient on cleaning the prosthesis/mucosal tissue interface. In fact, concave areas and long flanges could hinder proper hygiene access and promote biofilm accumulation.

Additionally, as proposed by Aparicio et al., a CBCT is recommended 1 year after surgery and every 5 years thereafter to evaluate the maxillary sinuses. As proposed by Aparicio et al., the Lund–Mackay staging system is suggested [18], which is a validated scoring system endorsed by the Task Force on Rhinosinusitis for research outcomes. This radiological assessment encompasses six distinct regions: anterior ethmoid, posterior ethmoid, maxillary, frontal, sphenoid, and the osteomeatal complex. Each of these regions is assigned a score of 0, 1, or 2. Any scan registering a score greater than 0 is indicative of an abnormal or “positive” result. In the context of zygomatic implant rehabilitation, intraoral X-rays, as suggested by Malevez et al. [19], may not yield significant information. This is primarily due to two key factors: Firstly, the atrophic maxilla often undergoes a reduction in the curvature of the palatal region. Secondly, zygomatic implants are typically placed at an inclined position. Furthermore, in cases where the head and a portion of the body of the implant are situated externally to the residual alveolar process or partially outside the anterior maxillary wall, these implants lack bone support around their entire circumference. In such instances, evaluating implant success by measuring marginal bone height becomes less meaningful since the implant was intentionally positioned, at least in part, beyond the osseous boundaries.

The proposed protocol for the follow-up and maintenance of patients rehabilitated with zygomatic implants consist of five steps that are described below, and it is suggested that it be used in the maintenance phase after the final prosthesis delivery (that is, approximately 4 months after surgery).

### 3.1. Step 1—Intra/Extraoral and TMJ Examination

**Figure 1:**
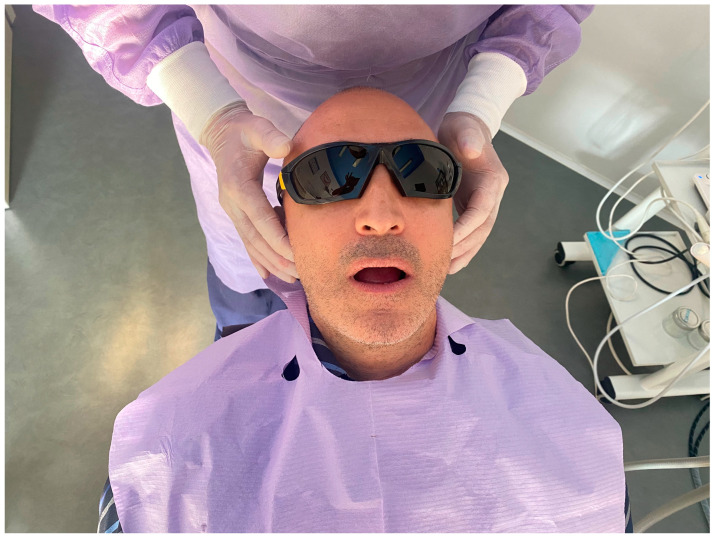
Examination of the TMJ in a patient with a full-arch rehabilitation supported by zygomatic implants.

The first step consists of the examination of intra- and extra-oral soft tissues and of the temporomandibular joint. The clinician should check the TMJ for clicking or referred pain (Figure 1). This is particularly important in patients rehabilitated with a full-arch immediate loading protocol, especially when the prosthodontic vertical dimension has been considerably changed. In case of TMJ problems, a careful examination of the dental occlusion balance is suggested, combined with electromyography through a portable Holter monitor capable of simultaneously recording the activity of the masseter muscles and the heart to evaluate episodes of bruxism. In case of bruxism, a night guard is suggested.

**Figure 2:**
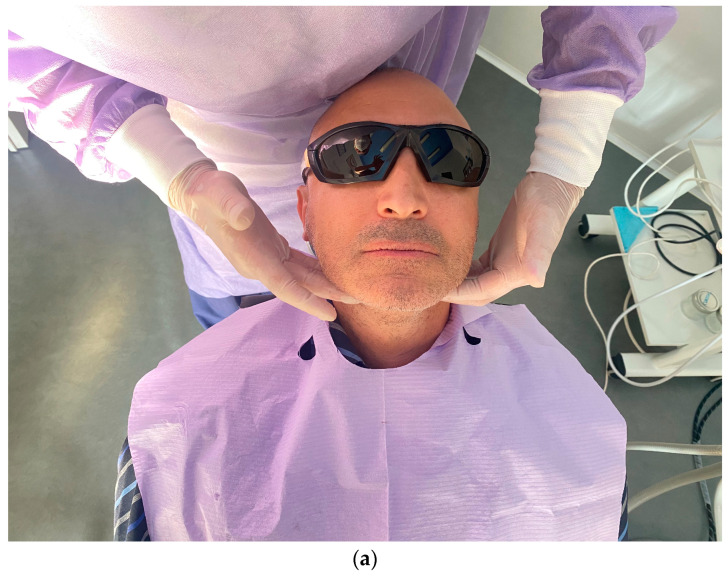

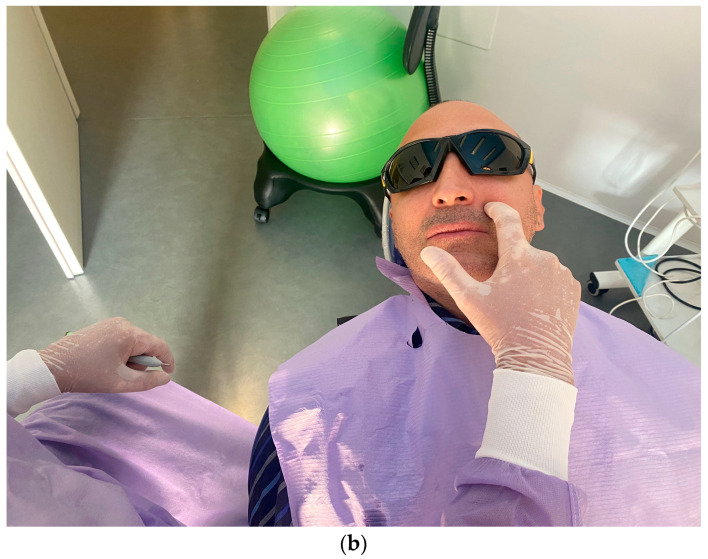
(**a**,**b**) Examination of the lymph nodes and of the sinuses.

The following steps consists of the palpation of facial and neck lymph nodes (Figure 2a) and the extraoral digital percussion of nasal sinuses (Figure 2b) in order to detect early signs of an inflammatory process around the implants and in the sinuses.

**Figure 3:**
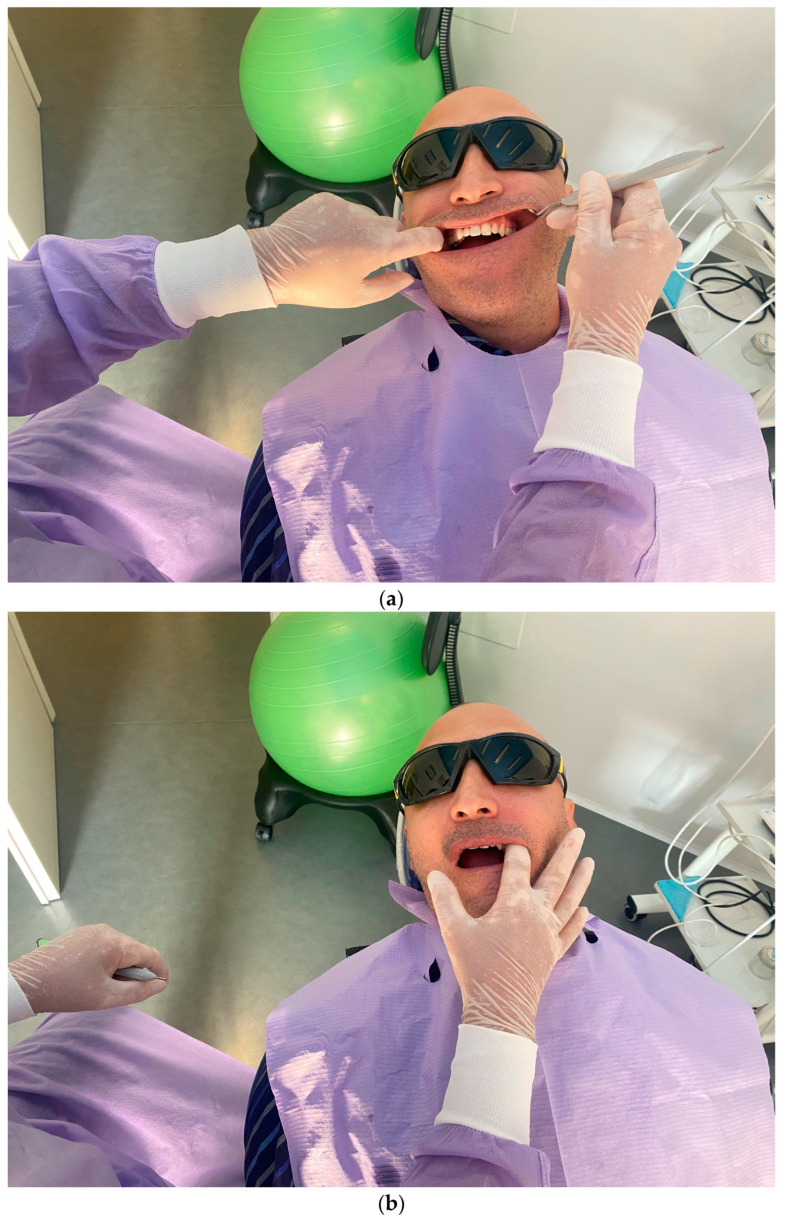
(**a**,**b**) Examination and palpation of the fornix.

Afterwards, intraoral soft tissues should be carefully checked to detect signs of masticatory trauma or infectious processes. This should include the examination of the tongue and of the general area, and the observation and palpation of the palate and masticatory mucosa. Additionally, an observation and palpation of the fornix to detect pain, the presence of fistulas, or suppuration must be performed (Figure 3a,b). Apical/coronal squeezing of peri-implant tissues should be conducted to detect the presence of exudate and/or suppuration.

It is also recommended to register persistent halitosis, as reported by the patients or perceived by the operator. This phase is particularly important to diagnose eventual rhinosinusitis. As suggested by Lanza and Kennedy [20], major and minor criteria to diagnose rhinosinusitis exist. Major criteria are facial pain or pressure, facial congestion or fullness, nasal obstruction, purulent discharge, hyposmia or anosmia, purulence on examination, and fever. Minor criteria are headache, fever (not acute), halitosis, fatigue, dental pain, cough, otalgia, or aural fullness. According to these criteria, the diagnosis of rhinosinusitis is made if two or more major criteria are present or one major and two or more minor criteria.

### 3.2. Step 2—Soft Tissue and Transmucosal Path Observation

**Figure 4:**
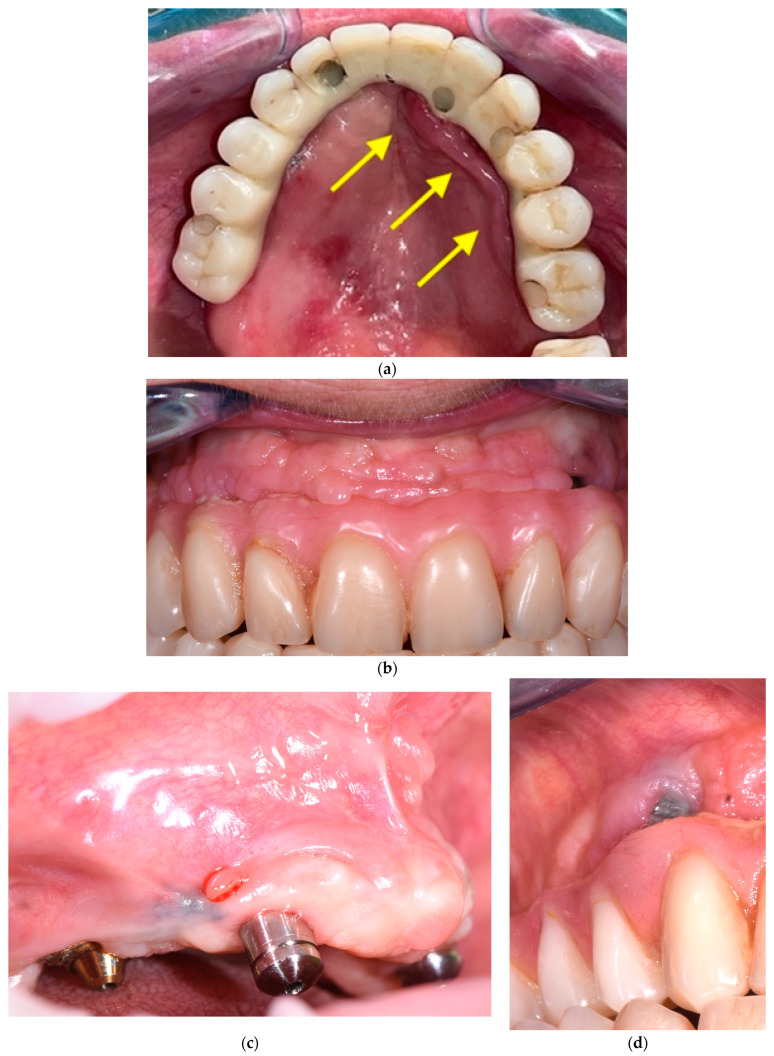
(**a**–**d**) Intraoral pictures of possible biological complications of zygomatic implants: decubitus (**a**), hypertrophy (**b**), fistulas (**c**), and dehiscence (**d**).

The second step consists of the examination of the soft tissues next to the implants and the prosthesis. In this phase, transmucosal decubitus (Figure 4a), fenestrations, hypertrophy (Figure 4b), fistulas (Figure 4c), and dehiscence (Figure 4d) must be identified and registered. In these cases, the prosthesis is unscrewed to assess peri-implant tissues and the morphology of the prosthodontic framework; investigations are conducted regarding any difficulties in maintaining hygiene due to inadequate space between the gums and the prosthesis. The devices to be used in this phase are retractors (i.e., Optragate, Ivoclar Vivadent, Schaan, Liechtenstein), gauze, and optical magnifiers.

### 3.3. Step 3—Peri-Implant Indices and Digital Stimulation of Tissues

For conventional dental implants, the latest guidelines define peri-implant probing as a necessary clinical procedure to assess crestal bone loss and determine the health or disease status around the implant [21]. However, the same criterion cannot be applied to extra-sinus zygomatic implants because it is impossible to monitor crestal bone that is not present on the vestibular aspect. Additionally, in zygomatic implants, the transmucosal path cannot be evaluated with a periodontal probe due to the lack of standard reference values, thicker mucosa, bulky prosthesis, and the use of angled abutments, which prevent crestal bone monitoring through probing.

Instead, the use of a probe around this type of implant could be employed for the clinical assessment of bleeding and the health status of the mucosal seal. The probe angle should be more open than the conventional 45° to avoid breaking the desmosomal seal surrounding the implants, which could potentially lead to an oro-antral communication [7].

**Figure 5:**
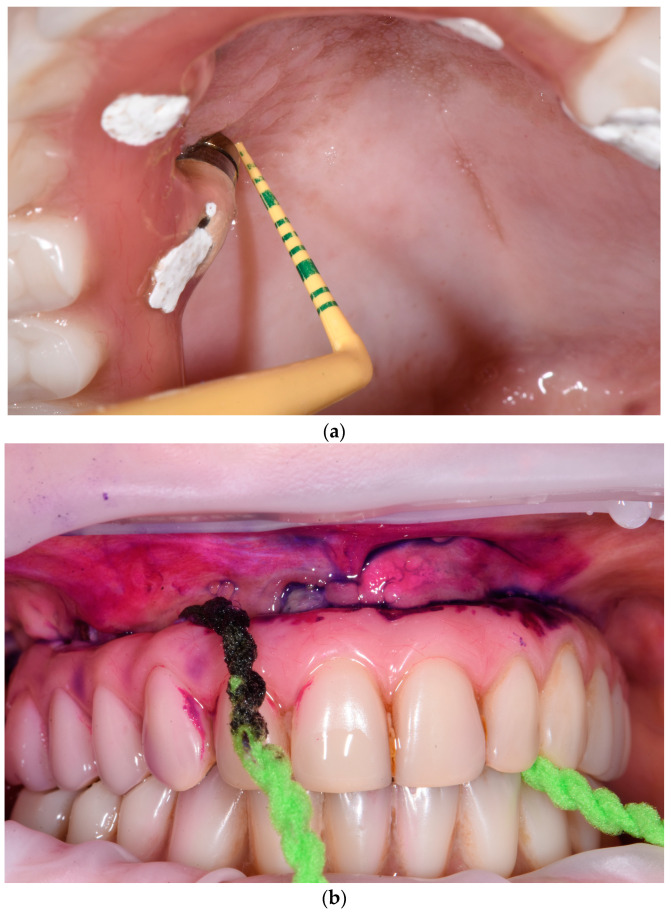
(**a**,**b**) Peri-implant probing and plaque check.

A gentle probing of 0.25 N is performed using a flexible, non-metallic periodontal probe (Figure 5a) to check the mucosal seal, record plaque index (PI), and any bleeding (BoP) and/or suppuration around the implants.

Plaque deposits on prosthesis and implants can be detected and shown to the patient using a plaque disclosing solution (Figure 5b). However, when dealing with acrylic resin prostheses, the use of a disclosing solution is recommended only if low-grit air-polishing systems (<65 µm) are available; otherwise, the removal of the disclosing solution may be challenging.

The devices used in this phase are a non-metallic flexible probe, plaque detector, spongy dental floss, and optical magnifiers.

### 3.4. Step 4—Prosthesis Examination

**Figure 6:**
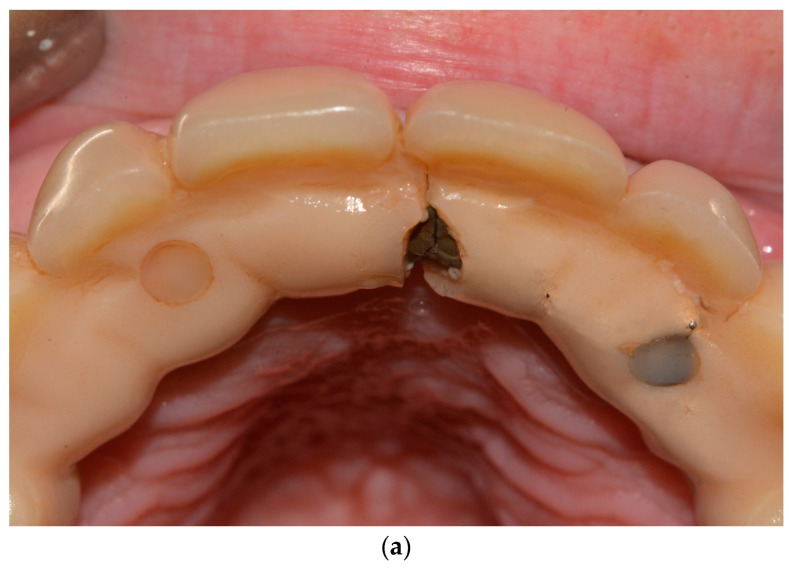

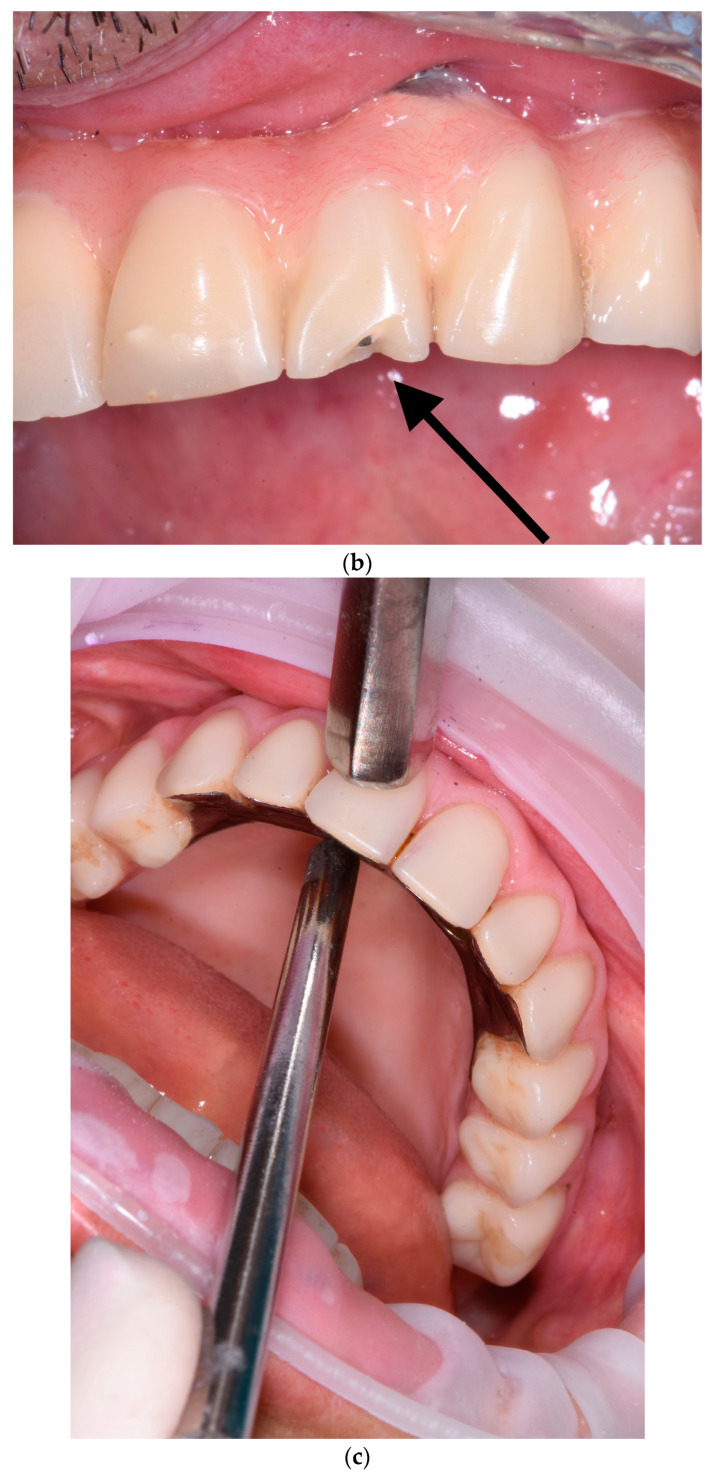

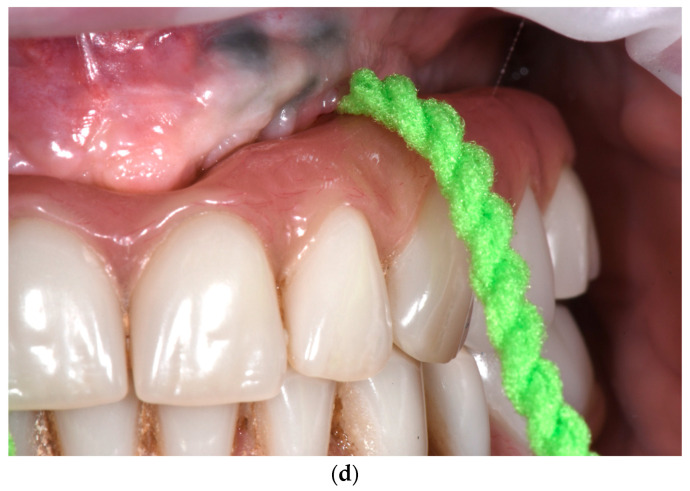
(**a**–**d**) Intraoral pictures of possible technical complications of zygomatic implants: chipping and occlusal wear (**a**), prosthesis fracture (**b**), vertical mobility (**c**), challenging access for hygienic devices (**d**).

Detecting mechanical issues during the first year of function of the implant-supported prosthodontic device is of utmost importance to prevent biomechanical complications that may compromise osseointegration and lead to inflammation in the peri-implant mucosal tissue. The healthcare provider overseeing the patient’s progress during this phase should possess the expertise to identify prosthodontic abnormalities and promptly communicate them to the specialist. In this perspective, the fourth step consists of the examination of prosthodontic structures to identify occlusal wear (Figure 6a), chipping (Figure 6a), fracture lines (Figure 6b), horizontal and vertical mobility (Figure 6c), and hygienic access to the prosthesis (Figure 6d). If occlusal problems are identified, then initially the opposing arch is assessed, and then the implant prosthesis is removed, followed by reassembly in an articulator to reassess masticatory movements. Repairs are carried out in case of chipping and, if necessary, palatal metal protection is added.

The materials used in this phase are spongy dental floss and optical magnifiers.

### 3.5. Step 5—Photographic Recording

The last suggested step is the collection of intraoral and extraoral pictures at least once a year to monitor the evolution of possible dehiscence, occlusal wear, and other complications.

## 4. Discussion

To the authors’ knowledge, this is the first paper presenting guidelines for the maintenance of patients rehabilitated with zygomatic implants and proposing a protocol to examine them. The only similar report available is the study by Aparicio et al. [16], where the ORIS criteria for the success of zygomatic-implant-supported rehabilitation are described. The objective of the Aparicio article was to examine the success criteria employed for both traditional and zygomatic implants and to present an updated Zygomatic Success Code outlining precise criteria for evaluating the results of a rehabilitation with zygomatic implants.

For more than three decades, the practice of bone grafting before or concurrently with implant placement has been a standard procedure in the oral rehabilitation of severely compromised patients. Despite a plethora of publications on the subject, the efficacy of sinus grafting procedures remains a topic of controversy [7]. Much of the existing literature that discusses these techniques lacks clearly defined criteria for determining implant success and failure, and frequently fails to provide initial bone height measurements along with standardized radiographic follow-up data [7]. A recently published retrospective paper reports a CSR of 95% at ten years and 85% at twenty years for implants placed simultaneously with lateral maxillary sinus floor augmentation [22]. At the same time, a systematic revision reports a CSR of 95.21% after 12 years for zygomatic implants [23]. The ultimate aim of zygomatic implants is to reinstate masticatory function, aesthetics, and comfort, and bolster self-esteem and social confidence in patients with severely compromised maxillae. Implant-supported fixed prostheses effectively fulfill all these goals, culminating in elevated patient contentment with the treatment and, subsequently, elevated success rates [24]. Studies analyzing patients’ quality of life and satisfaction report that revitalizing individuals with edentulous atrophic maxillae via fixed prostheses bolstered by a combination of zygomatic implants and anterior implants in the premaxilla led to a notably improved quality of life and heightened treatment satisfaction among patients [24].

The present manuscript highlights the challenges and possible complications associated with zygomatic implants. The authors emphasize the importance of maintaining a strict maintenance protocol and checking various aspects of intra- and extra-oral tissues to prevent possible complications.

The five-step approach proposed in this study aims at the early interception of complications and at maintaining the health of patients with zygomatic implants. The dental hygienist who eventually intercepts a complication must immediately submit the case to the surgeon in order to undertake the necessary checks and/or interventions.

The present manuscript represents a preliminary contribution to the development of detailed clinical protocols targeted at both professional and home maintenance of patients to improve long-term outcomes and patient satisfaction.

In fact, while specific devices and protocols have been proposed for the professional and home oral hygiene of full-arch fixed prostheses supported by standard dental implants [25], the same techniques might not apply to zygomatic implants, due to the specific features of peri-implant tissues and prosthesis design. For instance, air polishing devices have proven to be effective in the professional oral hygiene of fixed full-arch implant-supported prostheses, including when the prosthesis is bulky and is not removed.

However, they might not be safe if not properly applied, in the case of zygomatic implants, and specific guidelines for their use should be provided due to the delicate soft tissue seal of zygomatic implants and their proximity with other sensitive extraoral anatomic structures.

At the same time, some features of the present protocol could be used to analyze pterygoid implants. Pterygoid implant placement involves less invasiveness compared to zygomatic implant placement [26]. Pterygoid implants surpass conventional dental implants in length due to their requirement for insertion through the maxillary tuberosity and the pyramidal process of the palatine bone to securely engage with the pterygoid process of the sphenoid bone [27]. When employed in conjunction with the all-on-four technique, positioning a pterygoid implant within the posterior region of each maxillary quadrant eliminates the necessity for distal cantilevers. This extension of the posterior occlusion range facilitates comprehensive full-arch rehabilitation while simultaneously minimizing complications associated with the prosthetic design.

In a recent scientometric study paper, Ramal-Sanchez analyzed the research papers published within the interval 1990–2021 that included the keywords “zygomatic implants”. This study underscores the imperative of collaborative efforts among experts in this field to facilitate knowledge sharing. Such collaboration contributes significantly to standardizing this practice, thereby enhancing patients’ quality of life and mitigating potential complications effectively [28].

To promote clinical success and reduce the incidence of complications in the case of zygomatic implants, a surgical and prosthodontic learning curve is necessary but not sufficient, and should be combined with appropriate and dedicated maintenance programs. This cannot be prescinded from an effective collaboration of the entire dental team, and of the surgeon, prosthodontist, dental hygienist, and dental technician.

In conclusion, the present manuscript aims to provide guidelines to examine patients rehabilitated with zygomatic implants to detect complications that could undermine the implant-supported rehabilitation as soon as possible. Future reports should analyze the professional hygiene protocol to be used to maintain this kind of rehabilitation.

## Data Availability

No data is available.
